# May early intervention with high dose intravenous immunoglobulin pose a potentially successful treatment for severe cases of tick-borne encephalitis?

**DOI:** 10.1186/1471-2334-13-306

**Published:** 2013-07-03

**Authors:** Daniel Růžek, Gerhard Dobler, Hans Helmut Niller

**Affiliations:** 1 Academy of Sciences of the Czech Republic, Biology Centre, Institute of Parasitology, Branisovska: 31, CZ-37005 Ceske Budejovice, Czech Republic; 2 Department of Virology, Veterinary Research Institute, Hudcova: 70, CZ-62100 Brno, Czech Republic; 3 Department of Microbiology of the German Armed Forces, Neuherbergstr. D-: 11, D-80937 Munich, Germany; 4 Institute for Medical Microbiology and Hygiene, University of Regensburg, Franz-Josef-Strauss Allee: 11, 93053 Regensburg, Germany

**Keywords:** Arboviruses, T-cell, Inflammation, MRI, Macrophage, Neopterin, TBE, Tick-borne encephalitis, T2-weighted hyper intensity

## Abstract

**Background:**

Arthropod-borne viral encephalitis of diverse origins shows similar clinical symptoms, histopathology and magnetic resonance imaging, indicating that the patho mechanisms may be similar. There is no specific therapy to date. However, vaccination remains the best prophylaxis against a selected few. Regardless of these shortcomings, there are an increasing number of case reports that successfully treat arboviral encephalitis with high doses of intravenous immunoglobulins.

**Discussion:**

To our knowledge, high dose intravenous immunoglobulin has not been tested systematically for treating severe cases of tick-borne encephalitis. Antibody-dependent enhancement has been suspected, but not proven, in several juvenile cases of tick-borne encephalitis. Although antibody-dependent enhancement during secondary infection with dengue virus has been documented, no adverse effects were noticed in a controlled study of high dose intravenous immunoglobulin therapy for dengue-associated thrombocytopenia. The inflammation-dampening therapeutic effects of generic high dose intravenous immunoglobulins may override the antibody-dependent enhancement effects that are potentially induced by cross-reactive antibodies or by virus-specific antibodies at sub-neutralizing levels.

**Summary:**

Analogous to the increasing number of case reports on the successful treatment of other arboviral encephalitides with high dose intravenous immunoglobulins, we postulate whether it may be possible to also treat severe cases of tick-borne encephalitis with high dose intravenous immunoglobulins as early in the course of the disease as possible.

## Background

### Tick-borne encephalitis (TBE): epidemiology, radiologic findings and pathology

Together with other prominent human pathogenic arthropod-borne (arbo) viruses, such as yellow fever virus (YFV), dengue virus (DENV), Japanese encephalitis virus (JEV), West-Nile virus (WNV) and other less known relatives, the tick-borne encephalitis virus (TBEV) belongs to the *Flaviviridae* family (for review see [1]). Flaviviruses are enveloped viruses with a single-stranded RNA in positive-strand orientation. Three TBEV subtypes, namely the European, the Siberian and the Far-Eastern subtype, are endemic to different geographical areas in Europe and Asia. The Western TBEV subtype is mainly transmitted by the tick species *Ixodes (I.) ricinus*, while the Eastern subtypes are mainly transmitted by the tick species *I. persulcatus* from rodents and other small mammals. Vaccination is highly effective in preventing TBE disease and is recommended for residents exposed to TBEV within the endemic areas [2]. Firstly, after an incubation period of one or two weeks (4 to 28 days), the typical biphasic febrile disease involves a flu-like illness of four (1 to 8) days, allowing the virus to become viremic. Secondly, after a lag period of one week (1 to 33 days), an invasion occurs of the entire reticulo-endothelial system and the central nervous system (CNS). The virus is neurotropic and causes meningitis, meningoencephalitis, meningoencephalomyelitis or meningoencephalomyeloradiculitis, and may result in long-lasting or permanent neurological damage, known as post-encephalitic syndrome. In recent years, between 2,000 and 4,000 clinical cases were reported annually for Europe excluding regions of Russia and Asia [1,3]. Since mild flu-like disease occurs more frequently (an estimated 70 to 80% of cases) than neurological disease, the real case numbers are estimated to be much higher than the reported ones [3]. Full recovery takes place in slightly more than half of the reported clinical cases, while slightly less than half of the patients are afflicted with residual sequelae. The disease is fatal in less than 1% of European subtype TBE cases, but the lethality of the two Eastern TBEV subtypes is much higher [1]. In elder patients, severe disease courses are more frequent than in children, but severe courses also occur in children (for review see [4,5]). It has not been systematically resolved, whether antibody-dependent enhancement (ADE) may have taken place in some childhood TBE cases after post-exposure prophylaxis [6-8], as it has been described for secondary DENV infection. While enhancement was observed in cell culture [9], it has not been found in TBE-infected mice, after passive pre- or post-exposure prophylaxis had been given [10].

Widespread lesions in the CNS may involve gray matter and leptomeninges of the brain stem, medulla oblongata, nuclei, cerebellum and spinal cord (for review see [4,5]). A prominent perivascular infiltration by activated inflammatory cells such as macrophages and leukocytes is observed. Furthermore, neuronal degeneration, necrosis and neuronophagia occur. Elevated intrathecal neopterin that is secreted by stimulated macrophages indicates a high degree of macrophage and T-cell activation [11]. Magnetic resonance imaging (MRI) is reported as normal in the majority of cases [12]. However, enhanced signals in T2-weighted MRI scans have been regularly observed in the acute phase of severe TBE [13] and sometimes for extended time periods in protracted disease courses [6,8]. MRI studies demonstrated CNS damage mainly in the thalamus, cerebellum, nucleus caudatus and the brain stem [14].

The mechanisms by which TBEV causes encephalitis are not completely understood, but a composite of direct cytolytic viral damage and of considerable immune pathology is likely. Mouse models of TBE disease demonstrated that immune pathology contributed significantly to the neurological damage in TBE [15]. On the one hand, normal mice succumbed to the CNS inflammation caused by TBEV infection, while CD8-knockout mice, or mice with a severe combined immune deficiency (SCID), exhibited prolonged survival. On the other hand, the adoptive transfer of CD8^+^ T-cells to infected SCID mice significantly shortened their survival, while the transfer of CD4^+^ T-cells prolonged their survival. The viral load was the same in normal mouse strains and in CD8-knockout mice, while it was higher in SCID mice. The histological brain lesions were more moderate in immune suppressed mouse strains than in immunocompetent mice. The inflammatory infiltrates around meningeal vessels consisted mainly of CD8^+^ T-cells, and contained also histiocytes, but little CD4^+^ T-cells. These experiments proved a key role for the CD8^+^ T-cells in the pathology of TBE, and that TBE is essentially an immunopathological disease [15]. Moreover, experimental immune suppressive therapy with Cyclophosphamide led to an increased survival time of TBEV-infected mice [16]. The CNS pathology induced by other flaviviruses, such as WNV and Murray Valley encephalitis virus, was also more caused by the immune system than by cytolytic virus replication. Like in TBE, CD8^+^ T-cells were the main culprits for the immune damage caused by WNV, while CD4^+^ T-cells prolonged the survival of WNV infected mice [15,17]. Contrary to TBE, where the viral load was not correlated with the presence or absence of CD8^+^ T-cells, WNV load was lower in immunocompetent mice. Both in TBEV and WNV encephalitis, CD8^+^ T-cells were required for the eventual clearance of WNV from the organism and for mouse survival [15,18]. This shows that part of the neuronal damage is also directly due to virus infection [15,19]. Thus, the mouse experiments indicate that an immunomodulatory treatment that dampens the inflammatory immune response, while not disabling it, may help curbing flaviviral CNS pathology. A case in point was the rapid recovery of TBE patients from their clinical symptoms that were treated with the immunomodulatory antibiotic tetracycline that reduced an inflammatory response [20].

There is no specific treatment currently available for TBEV disease. Since immune modulation by tetracycline was helpful, immunomodulatory therapy may be the way to go [20]. Ideal therapy will (i) enhance protective immune reactions, (ii) normalize immune regulation, and (iii) suppress the damaging mechanisms by the immune response. A well-proven option of immune modulation is to administrate a generic high dose (1 to 2 grams per kilogram body weight) of intravenous immunoglobulins (IVIG) over a time course of 2 to 5 days. This is a similar treatment given for several acute hyper inflammatory autoimmune conditions, e.g. Kawasaki vasculitis, Guillain-Barré syndrome, myasthenia gravis, immune thrombocytopenia, macrophage activation syndromes, acute encephalitis, or may also be given as an adjunctive therapy for sepsis [21-24]. The best known effects of high dose IVIG are (i) blocking Fc receptors of macrophages, thereby occluding the clearance of antibody coated cells in the reticuloendothelial system, (ii) blocking the complement activation by abrogating the ability of aggregated antibodies to lead to complement activation, and through scavenging of complement components, (iii) specifically neutralizing pathogens by anti-pathogen antibodies present in the mixture. The mechanisms of reducing inflammatory cytokines, suppressing T-cell responses and dampening inflammatory reactions in general are not entirely clear yet [21-24].

## Discussion

### Case report collection

The neuropathological and neuroradiological findings of various arboviral encephalitides are similar in nature and therefore non-specific [13,25]. Therefore, it is interesting to note a series of case reports on successful intervention with high dose IVIG in severe cases of viral encephalitis of arboviral origin, other than flaviviruses and of flaviviral origin other than TBEV. However, to our knowledge, the early intervention with high dose IVIG in severe TBE cases has not been reported so far. The following case reports are grouped together with respect to the viral cause. An overview of the case reports is given in Table 1.

**Table 1:** Listing of case reports of viral encephalitis patients who were treated with high dose intravenous immunoglobulin (IVIG)

**Arboviruses**	**Encephalitis**	**Haemorrhagic fever**	**Refs.**
TBEV (flavivirus)	1		[26]
JEV (flavivirus)	2		[28-30]
DENV (flavivirus)		15	[31]
WNV (flavivirus)	15		[34-42]
EEEV (togavirus)	1		[44]
CHIKV (togavirus)	1		[45]
**other viruses**			
influenza A virus	2		[46,47]
herpes simplex virus	1		[48]
enterovirus	23		[49,50]
mumps virus	1		[53]

The outcome of IVIG-treatment was mostly favourable in the encephalitis cases. However, three of 15 WNV encephalitis patients died from their disease [34,38]. IVIG treatment had no influence on platelet recovery in 15 cases of secondary DENV haemorrhagic infection, but caused no adverse events either.

#### Tick-borne encephalitis virus

A 54 year old man had a severe and complicated TBE infection, after a complete vaccination against TBE with two boosters. He received the last booster 39 months before his disease. After two weeks the patient developed sinus tachycardia, hypertonia and gastrointestinal tract dysfunction. These symptoms and a fixed heart rate were taken as signs of autonomic failure. Late after disease onset, i.e. from day 20 to 24 of his illness, the patient received IVIG at a dose of 0.4 grams per kilogram body weight each day. While the dysautonomic symptoms improved and cardiac arrhythmia disappeared, the other neurologic symptoms did not change, and a post-encephalitic syndrome persisted that forced the patient into early retirement [26]. In this context, it may be interesting to note that actual IVIG preparations contain different levels of anti-TBE antibodies, depending on their geographical origin [27].

#### Japanese encephalitis virus

JEV is the most common cause of mosquito-borne flaviviral encephalitis in Asia. About 30,000 to 50,000 cases are reported annually. Contrary to TBE, mortality rate is high in the range of 25 to 30%. Sequelae are observed in about 50% of those who survive [28,29]. A 49-year-old Italian traveller to Vietnam with a severe form of JE showed extensive T2-weighted hyper intensities in a MRI scan. T2-weighted hyper intensities have also been described in other JE cases [25,30]. A diagnosis was established by detecting JEV-specific antibodies in the CSF and serum. The patient was treated symptomatically, but his condition worsened when on the sixth day of his disease a five day course of IVIG at a dose of 0.4 grams per kilogram body weight each day was started. It was not reported whether the applied IVIG preparation contained specific anti-JEV antibodies. Beginning with the next day, his condition started to improve. After three weeks he was discharged with slight symptoms of speech, motor and mental activity remaining, but with a normal MRI. Only a slight deficit in recent memory was observed one month after his discharge [28,29].

In addition to hypotension, respiratory failure and general meningoencephalitic symptoms, a 64-year-old man with JE had prominent signs of Parkinsonism. MRI scans showed T2-weighted hyper intensities in the thalamus and substantia nigra that correspond to signs of Parkinsonism. A more than four-fold increase of specific antibodies against JEV proved to be the cause of his disease. A total of 40 grams of IVIG containing anti-JEV antibodies was administered in two courses separated by one month. His symptoms ameliorated after each course. Although there were residual symptoms on discharge to rehabilitation, the treatment was thought to have saved the patient’s life [30].

#### Dengue virus

There are four subtypes of DENV and they are known for more severe disease courses upon secondary infection. In such cases, a frequently observed symptom is a pronounced thrombocytopenia leading to haemorrhage, most likely due to bone marrow infection and, in addition, possibly due to the clearance of thrombocytes through macrophages. More than 1,000 deaths occur worldwide annually due to dengue haemorrhagic fever and is associated with an increased level of platelet-associated IgG [31]. A randomized controlled study with 31 cases of secondary DENV infection in the Philippines compared the effect of early intervention with high dose IVIG versus non-intervention on platelet recovery. Beginning with the second day of their hospitalization, IVIG was given to 15 patients at a dose of 0.4 grams per kilogram body weight each day for a three-day course. It was not reported whether the IVIG-preparation (Gammamune, Bayer Health Care, Brea, California) contained anti-DENV antibodies. There was no effect on platelet recovery. The authors concluded that platelet clearance by macrophages through Fcγ-receptors is not the primary mechanism causing thrombocytopenia in this disease. However, an adverse outcome associated with the IVIG treatment was not observed in any of the IVIG-treated cases [31]. This indicates that the administered IVIG did not cause ADE, although enhancement has been shown to occur after secondary DENV infection, mainly with another subtype [2,31]. Dengue infection may also cause a variety of neurological complications which may result in poor recovery and long-term disability. It remains to be studied whether the rare case of DENV encephalitis or encephalopathy will respond to high dose IVIG. The conduction of randomized clinical trials investigating the potentially beneficial effects of IVIG for the various life-threatening manifestations of dengue fever has been proposed [32].

#### West Nile virus

WNV is originally endemic in East-Africa and the Middle East. Beginning with a couple of cases in New York City, between the years 1999 and 2002 WNV has conquered the USA through a bird-, horse-, mosquito- and human-cycle. WNV disease, including neurological involvement, has a high mortality in the range of 5 to 14% [33,34]. Eight cases of WNV encephalitis were treated with high dose IVIG at a dose of 0.4 grams per kilogram body weight each day for a five-day course. The blood used for this IVIG preparation (Omrix Biopharmaceuticals Ltd., Israel) was donated by healthy anti-WNV seropositive Israelis and, therefore, contained high levels of specific anti-WNV antibodies. Disease courses were severe, and four patients needed mechanical ventilation. Additionally, MRI scans were not systematically done. Six of the eight patients improved significantly upon the high dose IVIG treatment and were discharged, while two patients died from their disease. One of the patients who died had received a kidney transplant and was immune suppressed. The earlier the immunoglobulin treatment started, the faster was the improvement of the neurological symptoms. The two patients who died received high dose IVIG later than the fifth day of their disease. The authors concluded that the early administration of anti-WNV hyper immune high dose IVIG should be recommended for WNV encephalitis [34].

Acute flaccid paralysis is a life-threatening complication of WNV infection. A 55-year-old man with a quickly developing muscle weakness, reaching his respiratory muscles on the third day so that the patient needed mechanical ventilation was suspected to have a Guillain-Barré syndrome. Dexamethasone and plasmapheresis were administered. When on the sixth day WNV was suspected, corticosteroids and plasmapheresis were discontinued, and anti-WNV hyper immune high dose IVIG at 0.4 grams per kilogram body weight each day for a seven day course was started instead. This treatment led to the rapid improvement of his muscle weakness, so that the patient could be discharged to inpatient rehabilitation after one month [35]. Since the WNV diagnosis was established on the basis of an anti-WNV IgM in the serum, but IgG was not reported, it cannot be formally excluded that the IgM might have been a non-specific reactivity, since the acute flaccid paralysis might actually have had a different cause than WNV, although the diagnosis seems most likely correct.

There are additional cases of WNV encephalitis mostly successfully treated with anti-WNV hyper immune high dose IVIG [36-42], among them are several solid organ transplant patients [37,39-42]. A remarkable WNV case report including a review of the literature was presented by Rhee *et al*. (2011). The case report is unusual, because the infection was transmitted through a transplanted liver. When WNV was suspected in this case, serum was taken for analysis and the patient received 0.4 grams of anti-WNV hyper immune IVIG per kg body weight on the fourth and eighth day of his hospitalization and recovered fully. On the fifth day, a positive IgM, both in serum and CSF samples, were obtained before the start of IVIG, and the MRI scans pointed to WNV. Final proof came through a positive WNV-PCR from a retained blood sample of the liver donor who had not shown any WNV-typical symptoms at the time of his death through intracranial haemorrhage during a hypertensive crisis [41].

WNV with the highest number of case reports of arboviral encephalitis treated with high dose IVIG is special, because anti-WNV hyper immune IVIG preparations are available. This means that, besides the immunomodulatory effects of generic high dose IVIG which is dampening hyper inflammatory states without a noteworthy accompanying immune suppression, there is also a specific protective anti-WNV effect exerted by those immunoglobulin preparations in animal experiments [43].

#### Eastern Equine encephalitis virus

EEE is caused by EEE virus belonging to the *Togaviridae* family of the Alpha virus genus. EEE produces a mortality rate of about 30% and is the most severe of the mosquito-borne encephalitides. Poor outcome has been correlated with ages over 40, rapid progression to coma, severe hyponatremia, and a CSF white blood cell count above 500 at symptom onset [44]. A 69-year-old man with rapidly progressing EEE showed extensive T2-hyperintensities in repeated MRI scans and became comatose on the third day of his illness. On the third day of his hospitalization, one gram of methylprednisolon per day was started and slowly tapered. The diagnosis was established by identifying EEEV-specific antibodies in the CSF and serum. On the fifth day, the patient was given a total of 210 grams of generic IVIG over five days. It was not reported whether the applied IVIG preparation contained anti-EEEV antibodies. Beginning with the sixth hospital day, his condition started to improve. After more than two months, he was discharged home with few and relatively mild remaining symptoms, such as mild inattention, bradykinesia, short-term memory impairment, and emotional lability. All symptoms faded during the following year [44].

#### Chikungunya virus

CHIKV is a mosquito-borne virus belonging to the *Togaviridae* family of the Alpha virus genus. CHIKV typically causes a febrile disease in Africa and South-East Asia that is characterized by severe joint pain and can become haemorrhagic causing encephalitic symptoms in rare cases. The virus has been imported to Europe through international travel, and outbreaks have already occurred in Europe, e.g. in Northern Italy. Three cases reporting neurological symptoms were described by Chusri *et al*. (2011). The cases showed hyper intense signals in the MRI scan. Patient 1 was a 27-year-old woman who recovered on supportive measures. Patient 2 was an 85-year-old man who had not recovered during a follow-up one year later. Patient 3, a 44-year-old woman, who suffered a very severe form of late onset CHIKV encephalitis, was given a four day course of high dose IVIG and quickly recovered upon administration of the immunoglobulins and had recovered completely at a follow-up after six months. It was not reported whether the IVIG preparation contained anti-CHIKV antibodies [45].

#### Other viruses

High dose IVIG has also been reported to play an advantageous role in treating encephalitis induced by other viruses. Two severe cases of influenza A virus-induced encephalitis responded favourably [46,47]. Furthermore, a case of brainstem encephalitis induced by herpes simplex virus type I in an adult was effectively treated with a combination of high dose aciclovir, steroids and IVIG [48]. Early treatment of high dose IVIG has also been reported to lead to a negative viral load faster for 55 children with prior malignant disease who suffered severe enteroviral complications, including five cases of encephalitis. Thus, early intervention was recommended in order to improve the clinical outcome [49]. Another clinical trial with 18 confirmed cases of enteroviral encephalitis reported favourable clinical results after early intervention with one gram IVIG per kilogram body weight [50]. Acute disseminated encephalomyelitis (ADEM) is an autoimmune phenomenon that has a similar clinical appearance as viral encephalitis and may be triggered by infections or, in rare cases, by vaccinations. Essentially, the diagnosis of ADEM is based upon a combination of clinical and radiologic features and the exclusion of specific diseases that resemble ADEM. MRI scans again show disseminated T2-weighted hyper intensities [51,52]. There are three cases reported by Nishikawa *et al.* (1999) on the successful treatment of ADEM with high dose IVIG. Although in most cases of ADEM the trigger remains obscure, a mumps virus infection was found to be the cause for post-infectious ADEM in the third one of the described cases [53].

### Potential caveats

#### Adverse effects of high dose IVIG

The diverse case reports discussed above suggest that early intervention with high dose IVIG may be a general treatment option for viral and post-infectious encephalitides. Although IVIG is generally well tolerated, it is not free from side effects. Adverse effects are mostly mild that include aseptic meningitis, headache, fever, nausea, diarrhoea, blood pressure changes and tachycardia. More severe adverse effects include renal failure, thromboembolic events and anaphylactic reactions that are related to IgA deficiency. The prior exclusion of an IgA deficiency and the slow administration of IVIG in a sufficiently large liquid volume helps to minimize the serious side effects [24].

#### Antibody dependent enhancement (ADE)

With the exception of secondary DENV infection, ADE of flavivirus infection does not have any clear systematic *in vivo* proof and is still under debate. Although enhancement was seen in cultured macrophages or monocytes *in vitro*[9], it was not observed in TBE-infected mice [10]. Passive post-exposure prophylaxis for non-vaccinated persons was discontinued as a cautionary measure and never re-introduced again due to a couple of severe childhood TBE cases observed after administering specific hyper immune serum to non-vaccinated children after tick bites [6-8]. ADE of DENV infection of Fcγ-receptor bearing cells has been ascribed to cross-reactive non-neutralizing antibodies or to neutralizing antibodies at sub-neutralizing levels [2]. This may be due to the intermediate genetic distance of 60 to 75% at the amino acid level between the four DENV subtypes. Except for the tick-borne flaviviruses Powassan and TBE virus that have an intermediate genetic distance of about 75%, the genetic similarity between TBEV and the mosquito-borne flaviviruses is below 50% [1]. Thus, cross-enhancement should not occur between mosquito-borne flaviviruses and the TBEV. Even if ADE might be operative in TBE under rare circumstances, e.g. after prior infection with WNV [54] or after vaccination against YF, both of which is unlikely, or during infection at sub-protective levels of anti-TBE antibodies, which anecdotally may have been observed [6-8], these enhancing effects will most likely be overridden by the anti-inflammatory action of high dose generic IVIG. As a case in point, transiently immune suppressed mice were not harmed by the administration of specific anti-TBE antibodies. To the contrary, under transient immune suppression, serotherapy became more efficient and mostly prevented persisting infections [55].

## Summary

Active anti-TBEV vaccination continues to be the mainstay of TBE prophylaxis [2]. Even with an overall high vaccination rate, however, some TBE cases experiencing a severe disease course will remain. To our knowledge, early intervention with high dose IVIG has not been reported for TBE. A single severe TBE-case that we could find in the literature improved upon application of high dose IVIG late in the disease course [26]. Therefore, we wonder whether early intervention with high dose IVIG may pose a potentially successful treatment for TBE. However, also late intervention may be promising.

If this is the case, it may be important to obtain immunoglobulins from blood donors vaccinated against TBE [27], in analogy to anti-WNV hyper immune IVIG. Furthermore, clinical symptoms, CSF laboratory parameters including neopterin, together with a characteristic acute phase MRI may allow an early tentative diagnosis, even before serological test results are returned, and may indicate an early intervention with high dose IVIG. We propose to conduct a randomized controlled treatment study on severe TBE cases.

## Abbreviations

ADE: Antibody-dependent enhancement; ADEM: Acute disseminated encephalomyelitis; arbo: Arthropod-borne; CHIKV: Chikungunya virus; CNS: Central nervous system; CSF: Cerebrospinal fluid; DENV: Dengue virus; EEEV: Eastern Equine encephalitis virus; IVIG: Intravenous immunoglobulin; JEV: Japanese encephalitis virus; MRI: Magnetic resonance imaging; SCID: Severe combined immune deficiency; TBE: Tick-borne encephalitis; TBEV: Tick-borne encephalitis virus; WNV: West-Nile virus; YFV: Yellow fever virus.

## Competing interests

The authors declare that they have no competing interest.

## Authors’ contributions

DR, GD and HHN: wrote the paper; HHN: concept of the paper. All authors read and approved the final manuscript.

## Authors’ information

DR is Head of the Department of Virology of the Veterinary Research Institute and Research Scientist at the Institute of Parasitology, Academy of Sciences of the Czech Republic. GD is Head of the Department of Virology and Rickettsiology at the Bundeswehr Institute of Microbiology in Munich. HHN is Research Virologist and Senior Clinical Microbiologist in the Institute for Medical Microbiology and Hygiene at the University of Regensburg.

## Pre-publication history

The pre-publication history for this paper can be accessed here:

http://www.biomedcentral.com/1471-2334/13/306/prepub
